# ﻿Broad-ranged, highly disjunct, locally rare and severely endangered: the challenging risk assessment and a global conservation strategy for *Ericasicula* Guss. *sensu lato* (Ericaceae)

**DOI:** 10.3897/phytokeys.253.121945

**Published:** 2025-03-17

**Authors:** Salvatore Pasta, Hicham El Zein, Ozan Şentürk, Salih Gücel, Leopoldo de Simone, Bertrand de Montmollin

**Affiliations:** 1 National Research Council (CNR), Institute of Biosciences and BioResources (IBBR), Palermo, Italy Institute of Biosciences and BioResources Palermo Italy; 2 IUCN/SSC/Mediterranean Plant Specialist Group, Neuchâtel, Switzerland IUCN/SSC/Mediterranean Plant Specialist Group Neuchâtel Switzerland; 3 Centro Conservazione Biodiversità (CCB), Department of Life & Environmental Sciences, University of Cagliari, Cagliari, Italy University of Cagliari Cagliari Italy; 4 Royal Botanic Garden Edinburgh, Edinburgh, UK Royal Botanic Garden Edinburgh Edinburgh United Kingdom; 5 Faculty of Science, Ege University, Bornova-İzmir, Turkiye Ege University Bornova-İzmir Turkiye; 6 Environmental Research Center, Near East University, Nicosia, Cyprus Near East University Nicosia Cyprus; 7 Department of Life Sciences, University of Siena, Siena, Italy University of Siena Siena Italy

**Keywords:** Biogeography, cliff vegetation, conservation biology, ecological field surveys, international partnership, red listing, unmanned aerial systems

## Abstract

The distribution range of *Ericasicula* Guss. *sensu lato* spans the central and eastern Mediterranean Basin, but shows a significantly fragmented pattern, and its populations are locally subject to multiple threats inducing continuous regression. The species is distributed across five countries, Italy, Libya, Cyprus, Türkiye and Lebanon, and includes two subspecies, subsp. sicula and subsp. bocquetii, currently represented by 31 and 8 stands, respectively. This study provides an updated overview of the distribution, ecology, and conservation status of both subspecies. New distribution data and ecological information were gathered through fieldwork, literature, and herbarium specimens. In Sicily (Italy), unmanned aerial systems and high-resolution digital elevation models were employed to perform a detailed census of the last extant stand, mapping its distribution and calculating its 3D occupation surface. Based on our analyses, *Ericasicula* is evaluated as Least Concern (LC) at the global level, even though each subspecies and subpopulation are nationally endangered. In fact, 19 locations of E.siculasubsp.sicula were not confirmed recently, and this subspecies should be considered as Critically Endangered (CR) in Italy and Vulnerable (VU) in Lebanon, Cyprus and Türkiye. In Libya, E.siculasubsp.sicula is VU due to severe habitat degradation. E.siculasubsp.bocquetii, formerly known from a few locations in the mountains of SW Anatolia, Türkiye, has been found at lower altitudes in several new locations and is also assessed as VU. Further fieldwork is recommended to better assess the demographic trends of the different subpopulations. Genetic analyses are needed to clarify the taxonomic value of infraspecific taxa previously described and to guide future conservation efforts of the most unique and genetically rich stands, both in-situ and ex-situ. Improving the conservation strategies for taxa like *Ericasiculas. l.* requires the collaboration of specialists from all involved countries, making it crucial to maintain networks of experts in the Mediterranean.

## ﻿Introduction

### ﻿Biogeographic interest of the heathers of *Ericasicula* group

Extreme geographical disjunction is a very rare and fascinating phenomenon among Mediterranean plants living in vertical environments. In fact, very disjunct subpopulations usually have been isolated long enough to have differentiated into distinct species (Davis 1951). Nevertheless, there are notable examples of significant disjunction, particularly among Paleogene relict species which did not evolve into distinct species despite their populations being presumably separated for a long time, such as isolated populations of *Zelkovaabelicea* on the Cretan Mountains (Christe et al. 2014), or the strikingly disjuct populations of *Rhododendronponticum* in the Iberian Pensinsula and Georgia (Mejías et al. 2006). The large majority of these vulnerable taxa persist only in habitats that provide environmental conditions enabling their survival, such as north-facing cliffs and sheltered gulleys, where anthropogenic disturbance (e.g., overgrazing, fire) is low. These areas also experience reduced interspecific plant competition and lower drought stress due to particularly favourable microclimatic conditions (Thompson 2020).

In some cases, differentiation among disjunct populations of a given species led to the description of taxa that are formally recognised at the varietal or subspecific level, as is the case in *E.sicula*. These heathers form a rather distinct and isolated group (Ojeda et al. 1998), nested within *Erica* (McGuire and Kron 2005; Mugrabi de Kuppler et al. 2015; Pirie et al. 2024), and in the past they were classified under a distinct genus, *Pentapera* (Klotzsch 1838) due to their pentamerous flowers, in contrast to the tetramerous flowers of all other *Erica* species of the world. However, most scholars have argued against recognising this group of heathers as a separate genus or subgenus, in part due to the lack of other strikingly distinctive traits (see Bentham 1839; Boissier 1875; Bentham and Hooker 1876; Drude 1897; Webb and Rix 1972; McClintock 1980 and 1989; Meikle 1985 and, more recently, Nelson 2011; Elliott et al. 2024; Oliver et al. 2024).

Variation within *E.sicula* may be significantly influenced by local abiotic factors, such as substrate and climate, as well as disturbances. A taxonomic synthesis of the epithets referring to the *Ericasicula* group is presented in Table 1.

**Table 1.** Taxonomic and synonymic prospects of the epithets referring to the *Ericasicula* group. In bold: accepted names according to POWO (2025) and adopted in this paper.

***Ericasicula* Guss., Cat. Pl. Hort. Boccadifalco: 74. 1821 (subsp. sicula)**
*Pentaperasicula* (Guss.) Klotzsch, Linnaea 12: 498. 1838
*Ericagussonei* Schouw (unpublished manuscript)
Pentaperasicula(Guss.)Klotzschvar.libanotica Barb.-Boiss. & Barbey, Herb. Levant: 144. 1882
EricasiculaGuss.var.libanotica (Barb.-Boiss. & Barbey) Holmboe, Stud. Veg. Cyprus: 142. 1914
Pentaperasicula(Guss.)Klotzschsubsp.libanotica (Barb.-Boiss. & Barbey) Yalt., Notes Roy. Bot. Gard. Edinburgh, 28: 13. 1967
EricasiculaGuss.subsp.libanotica (Barb.-Boiss. & Barbey) P.F. Stevens, Fl. Türkiye & East Aegean Islands, 6: 97. 1978
EricasiculaGuss.subsp.cyrenaica Brullo & Furnari, Webbia, 34: 164. 1979
*Pentaperabocquetii* Peșmen, Candollea, 23. 271. 1968
*Ericabocquetii* (Peșmen) P.F. Stevens, Fl. Türkiye 6: 97. 1978
**EricasiculaGuss.subsp.bocquetii (Peșmen) E.C. Nelson, Hardy Heathers: 299. 2011**

**Figure 1.**
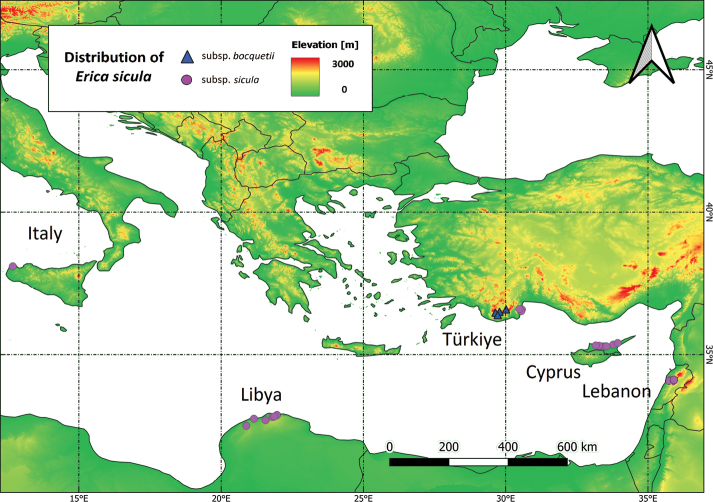
Distribution range of the two subspecies of *Ericasicula* Guss.

An additional aspect of interest in this puzzling study case is the extremely small area occupied by the stands of these taxa in each country (Fig. 1). Apart from the Italian one, all other subpopulations of E.siculasubsp.sicula are situated in countries of the eastern Mediterranean. The subpopulations growing in Lebanon, Cyprus and SW Anatolia were previously considered as a separate taxon, E.siculasubsp.libanotica (Yaltırık 1967; Browicz 1983). Similarly, the subpopulation of N Libya has been referred to as subsp. cyrenaica (Brullo and Furnari 1979). Concerning E.siculasubsp.bocquetii (Peşmen 1968), it is documented to occur in the Antalya region in W Taurus (SW Anatolia, Türkiye).

The minimum geographic distances between the populations are remarkable (Fig. 1): c. 850 km between SW Anatolia and N Libya, c. 1000 km between NW Sicily and N Libya, c. 1600 km between NW Sicily and SW Anatolia, c. 300 km between SW Anatolia and N Cyprus, c. 550 km between SW Anatolia and Lebanon and c. 250 km between N Cyprus and Lebanon.

### ﻿Aims of the paper

Based on data gathered from the literature, herbarium records and field surveys, we present an updated synthesis of the distribution and ecology of the known populations within the *Ericasicula* group. Additionally, we assessed the extinction risk of both subspecies at global and national scales, identifying key knowledge gaps and outlining critical conservation actions required to enhance the status of extant populations.

## ﻿Methods

Researchers from four of the five countries where these taxa are found collaborated on this study. We analysed and combined historical and recent first-hand field data to trace the past and present distribution and to clarify the ecological requirements of the different populations of *Ericasiculasensu lato* (*s.l.*).

### ﻿Literature survey

To begin, we meticulously analysed the extensive scientific and horticultural literature available on these taxa, including regional floras, such as the floras of Lebanon (Mouterde 1983), Syria, Palestine, and Sinai (Post and Dinsmore 1932), Turkey and the East Aegean Islands (Stevens 1978), Cyprus (Meikle 1985) and Europe (Webb and Rix 1972).

### ﻿Herbarium specimens

We then examined the specimens preserved in various European herbaria (AMD, B, BM, BOLO, C, CAT, COI, E, FI, G, K, L, LD, LM, MHNF, NAP, P, PAL, PAV, PI, RO, S, W, WAG, WSY), as well as in the non-European herbaria of the countries where the taxa are found, namely Cyprus (NEUN), Lebanon (BEI), Libya (ULT) and Türkiye (AEF, AKDU, ANK, EGE, GAZI, HUB, ISTE, ISTF, NGBB). The acronyms of the above listed herbaria follow the standard abbreviations of the Index Herbariorum (Thiers 2025). We also referred to the national database of Turkish master’s and doctoral theses (Republic of Türkiye, Council of Higher Education, Thesis Center, https://tez.yok.gov.tr/UlusalTezMerkezi/giris.jsp) to gather occurrence data related to the Antalya province. Additionally, we compiled an inventory of specimens kept in the European herbaria. Geographic coordinates were assigned wherever possible to facilitate mapping of both literature records and dry specimens.

### ﻿Field work

Field surveys were conducted in Italy, Lebanon, Türkiye and Cyprus to revisit historical localities where the species had previously been reported, to discover new occurrences in potential habitats, to accurately map its distribution, to collect additional information on its ecology and threats, and to accurately assess extinction risk. In Italy, we utilised the data previously collected between 2017 and 2020 by one of the authors (LDS) from the sole remaining locality where E.siculasubsp.sicula occurs, namely Mt. Cofano (de Simone 2020). Additional explorations in the neighbouring mountains were carried out between 2019 and 2022. In Lebanon, the Nahr Ibrahim Valley, or Adonis River, where the taxon occurs, was thoroughly surveyed by one of the authors (HEZ) in 2022 and 2023. In Cyprus, fieldwork was conducted in 2020 by one of the authors (SG) through the Kyrenia mountain range to map the entire extent of occurrence of the species on the island. In Türkiye, several localities with E.siculasubsp.sicula were visited and confirmed in the province of Antalya. As for E.siculasubsp.bocquetii in Türkiye and E.siculasubsp.sicula in Libya, fieldwork could not be conducted; therefore, herbarium specimens and literature data were used, with the most recent records being considered. For all countries, we deemed it appropriate to map the localities of unconfirmed historical records. The names of the localities as well as the information provided on the specimen labels were used to estimate their position on the maps. However, risk assessments were performed based only on confirmed and extant stands.

### ﻿Unmanned aerial systems

During field activities, in Italy it was also possible to exploit unmanned aerial systems (UAS) to perform a detailed census of its only extant stand. Using UAS-derived 3D models of target areas where the species was present and high-resolution digital elevation models of the entire study area, it was possible to perform a detailed census of the stand, map in detail its distribution and calculate its 3D occupation surface.

We carried out separate aerial surveys for each sampled area in spring 2019. We used a DJI Phantom 4 (hereafter drone) along vertical transects parallel to the cliff. The drone has a built-in GPS and a gimbal mounting a 12 MP camera that was manually triggered by a remote radio controller. The drone never exceeded a Ground Sampling Distance of 0.3 cm/pixel. Prior to the flight, two to four ground control points (GCPs) were positioned on the cliff wall. A Bosch laser distance meter (Bosch GLM 50) was used to measure the distance between GCPs centres.

Following the pipeline documented in de Simone (2020), the geo-referenced photos were used for the creation of a point cloud and a 3D model. For this purpose, the dataset was processed using Agisoft Metashape (Agisoft LLC, St. Petersburg, Russia). The general workflow suggested in the software user manual was followed. Photo alignment was performed using “high” as accuracy settings. The resulting point cloud was manually cleaned from homologous points and artefacts.

Subsequently, the GCPs positioned on the cliff were manually marked in each photo, and their distance was added to the software as a scale bar-based information for optimizing the results of photo alignment. Both the dense cloud and 3D model were performed using “medium” quality settings. Then, the whole photo dataset was manually scrutinized to detect each individual of E.siculasubsp.sicula. When an individual was detected, we marked its spatial position by placing a marker (i.e., a point of the dense cloud, with its X, Y, Z embedded information) on its centroid. The photo identification of E.siculasubsp.sicula was straightforward because it was in full bloom and literally covered with pale pink-white flowers. For logistic reasons, UAS could not be used in Cyprus, Türkiye or Lebanon.

### ﻿Mapping

Spatial data were all mapped using the software QGIS (version 3.28). Geographic coordinates, provided in decimal degrees using the WGS84 coordinate system (EPSG: 4326) were made available on the GBIF platform (https://doi.org/10.15468/8dk2qz) and will therefore be accessible to users in future updates of the Erica Identification Aid (Oliver et al. 2024).

### ﻿Extinction risk assessment

The extinction risk for each subspecies of *E.sicula* was assessed separately in each country according to the International Union for Conservation of Nature Red List categories and criteria (IUCN Standards and Petitions Committee 2024). The Extent of Occurrence (EOO) was calculated as the minimum convex hull based on the taxon occurrence records. The Area of Occupancy (AOO) was calculated using a cell width of 2 km, equivalent to a 4 km^2^ grid cell. When existing, previous assessments were reviewed and updated, as it was the case for Italy (Domina et al. 2012) and Lebanon (Stephan et al. 2017). Finally, the comprehensive global assessment of both taxa of the *E.sicula* group was carried out by consolidating the data provided by each national assessment.

The terms ‘population’ and ‘subpopulation’ are used according to the definitions provided by IUCN (2012). More specifically, the populations of both subspecies encompass all stands where these taxa are found, while subpopulations are defined as geographically or otherwise distinct groups in the population (characterised by minimal demographic or genetic exchange, typically no more than one successful migrant individual or gamete per year; we do not currently have data to confirm this).

## ﻿Results

### ﻿Morphological variability within the *Ericasicula* group

We collated all available information on the morphology, ecology and biology of the two subspecies from the literature. The main distinctive morphological traits among the populations of the *E.sicula* group are summarised here below (Table 2).

**Table 2.** Summary of the intra- and inter-specific morphological and phenological variability within the *Ericasicula* group.

**Figure 2.**
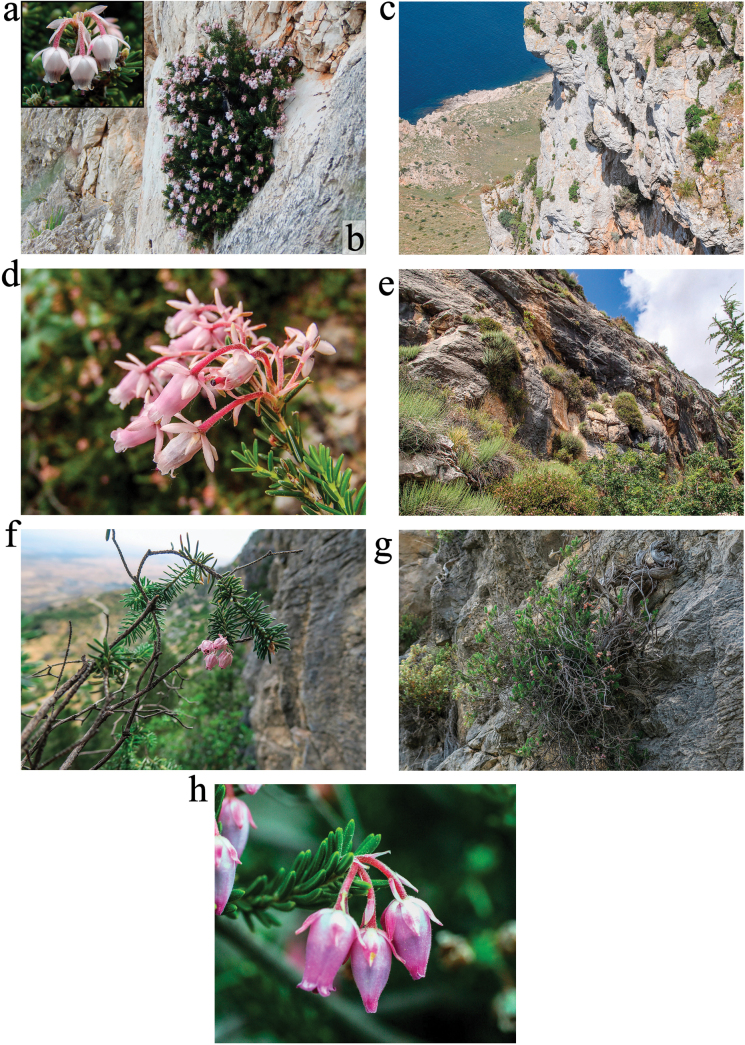
Ericasiculasubsp.sicula in its type locality, Monte Cofano (NW Sicily, Italy): flowers and overall habit (**a, b**: photo credit D. Salemi) and habitat (**c**: photo credit L. de Simone); E.siculasubsp.sicula at Aaqoura, Byblos District, Lebanon: flowers and habitat (**d, e**: photo credit: H. El Zein); E.siculasubsp.sicula at Buffavento, Cyprus: flowers and habitat (**f, g**: photo credit S. Cambria); E.siculasubsp.bocquetii at Kaş, Province of Antalya Türkiye: flowers (**h**: photo credit: Z. Aytaç).

**E.siculasubsp.sicula**
Sicilian (Italian) subpopulation (Figures 2a-c): erect 20-60 cm tall cushion-shaped shrub, with robust woody erect to ascending branches; the young twigs are subterete, densely pubescent in their upper side due to small patent glandular hairs; evergreen leathery leaves densely pubescent when young, linear, patent, 1 × 5-6 mm, in whorls of 4(-5), glossy dark green above, whitish beneath, with incompletely revolute and contiguous margins, concealing the lower surface forming a whitish line; flowers actinomorphic, 5-merous, grouped in terminal umbels of 4-6(10); pedicels 2 × 4 mm with 3 pink lanceolate bracteoles near the middle; 5 sepals ovate-lanceolate, pubescent, pink, 4 mm (2/3 of the corolla); 5 petals entirely welded forming a white or very pale pink urceolate, sparsely pubescent corolla, 4 × 6-8 mm; stamens 10, dark purple, anthers without appendages, included; ovary 5-locular, pubescent; stigma capitate (from Pignatti 2018). Plants start to flower in December, but the usual blooming period begins in February; its acme is between April and May and lasts until June (LDS, pers. obs.), whilst the fruits ripe during the summer season. The blooming time of the congeneric *Ericamultiflora* L., often co-occurring in close vicinity with *E.sicula*, is autumn (September to November), hence the two heathers are probably visited by different pollinators (LDS, pers. obs.).
Lebanese subpopulation (Fig. 2d, e): according to Mouterde (1983) [*Erica] sicula* is a shrub, 30-150 cm or more, very branched, with young branches pubescent and slightly velvety leaves, whorled in fours, often very deciduous during drying. Flowers in small umbels of 4-5, terminal, fairly large, borne on pedicels with three bracts, longer than the calyx. Sepals lanceolate, obtuse, slightly tomentose, three times shorter than the tomentose corolla itself. Corolla with lobes shorter than the tube, folded. Ovary white, tomentose. Mouterde (1983) adds these distinctive traits for var. libanotica: the entire plant is glabrous, except for the pedicels, which are puberulent; the terminal flowers are often more than five, and also lateral flowers are present, either solitary or in short fascicles. Flowering takes place all year round, except possibly from June to November.
Turkish subpopulation: according to Stevens (1978), local plants, previously referred as to E.siculasubsp.libanotica, would differ from those of the type (Sicilian) locality for several traits, like the size of the individuals (up to 150 cm), their almost glabrous twigs, the longer and narrower leaves (8.5-13 × 0.8-1.3 mm), the lack of a whitish mucrone on the leaf tip, the bigger and less hairy flower parts (pedicels 7-15 mm, sepals 6.5 mm, petals 7.5-9 × 5-5.5 mm) and the colour of the corolla (usually bright pink instead of pale pink-white).
Cypriot subpopulation (Figures 2f-g): local plants are smaller than the Turkish ones, probably because of stressful conditions induced by overgrazing and frequent fire disturbance. According to Viney (1994): “spreading shrub to 60 cm with tough, brown, flaking stems, needle leaves c. 7 mm long with edges rolled over, in close whorls of 4; flowers in umbels of 3-8 or more at the end of branches; corolla in various shades of pink (even to white), 7-8 mm long, bell-shaped-cylindrical, with 5 tiny-ended lobes; dark purple anthers inside the bell, but style shortly protruding. Flowering: March-July” (until August according to Meikle 1985).
Libyan subpopulation: according to Brullo and Furnari (1979), who described these plants as belonging to E.siculasubsp.cyrenaica, the plants growing in Cyrenaica differ from the type for their dwarfed habit, mostly growing as subshrubs (but Trotter 1915 observed well-developed individuals reaching 1 m), and for having 4.5-5 × 1.5-1.8 mm sepals, a 6 mm long corolla inflated in the lower part and evidently constricted in the upper one, the teeth up to 1 mm long, stamens 5 mm long, anthers 1.5-1.8 mm long, styluses 4.5 mm long hairy only in the upper part and rarely in the lower one.
**E.siculasubsp.bocquetii**
Small, 25-40 cm high, irregularly branched shrub that differs from the other taxa of the group for its overall dwarfed size: the flowers are smaller (Fig. 2h), the leaves are 3-4(-5) mm long, typically arranged in whorls of 3 rather that 4, and anthers lack spurs. The terminal inflorescence bears 1-4 purplish flowers, reduced leaves at the base (pedicels 8-13 mm, bracts and bracteoles 1.3-2 mm). Sepals 3-4 mm. Pale purple urceolate corolla 5-6(8)3 × 2-4 mm, somewhat pubescent. Filaments 2.4-4 mm, bent; anthers 2-2.5 mm, impressed styles 3.5-4 mm. Seeds compressed ovoid 0.5 × 0.3 mm (Peşmen 1968; Yaltırık 1971; Stevens 1978).

Table 3 provides a list of the available drawings related to *Ericasiculas.l.* These illustrations help to better understand the morphological characteristics and habitus of the plants in question.

**Table 3.** Inventory of the available iconographic sources concerning the *Ericasicula* group (in chronological order).

**Ericasiculasubsp.sicula**
Gussone, Ic. Fl. Sic. T. 197 (not found, Authors’ note)
C.F. Schmidt in Klotsch (1841-1844): table 19
http://plantillustrations.org/illustration.php?id_illustration=187541&SID=0&mobile=0&code_category_taxon=2&size=0
M. Smith in Hooker (1888): table 7030
http://plantillustrations.org/illustration.php?id_illustration=4516&SID=0&mobile=0&code_category_taxon=2&size=0
J. Weathers (1874-1955): fig. 45, p. 335
http://plantillustrations.org/illustration.php?id_illustration=384340&SID=0&mobile=0&code_category_taxon=2&size=0
Fiori and Paoletti (1899-1904): fig. 2649, p. 312
Lojacono-Pojero (1907): fig. 1, plate I
Trotter (1915) fig. 91, p. 243
Siddiqi (1978): fig. 2, p. 5
A. Zizza in Brullo and Furnari (1979): fig. 7, p. 165 (as Ericasiculasubsp.cyrenaica)
Mouterde (1983), Plate II, n° 4 (as *Pentaperasicula*)
Nelson (2011), figs. A-T, p. 298
**Ericasiculasubsp.bocquetii**
Peşmen (1968): fig. 3 a and c, p. 272 (as *Ericabocquetii*)

### ﻿Past and present distribution of *Ericasiculas.l.*

To provide a clear and updated distribution map of the stands in each country, we compiled a comprehensive list in Suppl. material 1. It contains detailed information retrieved from the literature and from the labels of the 212 herbarium specimens examined, such as the names of the collection localities, collectors, dates, specimen numbers, the herbarium where the specimen is housed, bibliographic references, geographic coordinates (estimated or provided by the sources), as well as the information on the stands observed or collected during recent field surveys. Nearly half of the specimens of E.siculasubsp.sicula were collected in Italy (88), followed by Libya (35), Türkiye (25), Cyprus (22) and Lebanon (20), while E.siculasubsp.bocquetii accounted for 22 specimens. Field surveys performed in Italy, Lebanon and Cyprus confirmed most of the historical occurrences. However, two of the three historical stands reported for Sicily, along with two from Lebanon and four from Cyprus, became extinct. Additionally, field surveys carried out in Türkiye during spring 2024 (i.e. after the submission of the first version of the manuscript) allowed the discovery of two stands of E.siculasubsp.sicula very close to historical collection sites. Detailed information for each country is provided in the following text.

#### ﻿Italy (and Malta)

**Figure 3.**
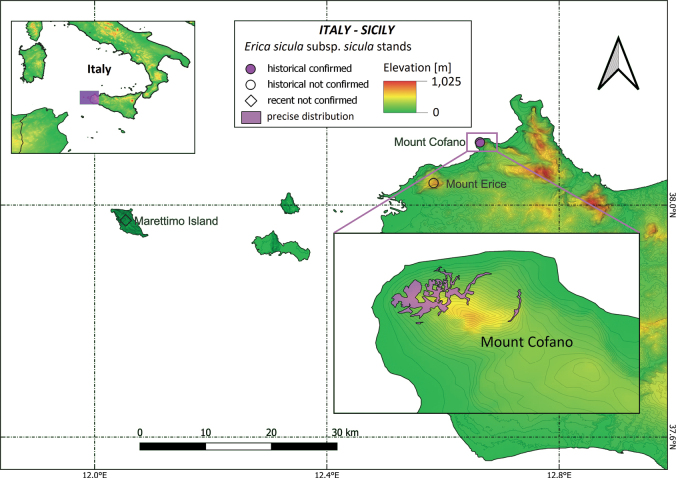
Past and present distribution of Ericasiculasubsp.sicula in Sicily (Italy) based on literature data and herbarium specimens; below: detailed distribution on Mt. Cofano (from de Simone 2020, modified).

The Sicilian subpopulation of E.siculasubsp.sicula currently occurs only on Mt. Cofano in NW Sicily, where it was first observed growing on its N-facing cliffs (“al Crocefisso”: Gussone 1821; “al Passo della Zita”: Ponzo 1900). Claims about the occurrence of other NW Sicilian stands date back to the early XIX century and have never been confirmed afterwards (Fig. 3). On Marettimo Island it was reported as extremely rare by Gussone (1832, 1843) with no further details on the exact location of the stand. No specimen has been found to confirm its past occurrence on the island, where it seems to have long since disappeared (Francini and Messeri 1956; Scuderi 2003; Gianguzzi et al. 2006; Gianguzzi et al. 2011; Domina et al. 2019; LDS, pers. obs.). However, Pignatti and Pignatti-Wikus (1990) reported observing it on a north-facing cliff below the summit of Pizzo Falcone, and Osti (2009) also claimed to have seen it on the island. It was observed on Monte Erice solely by Gasparrini during his field collections in Sicily between 1828 and 1831 (Alippi Cappelletti 1999). Its past presence there, reported by Lojacono-Pojero (1907) but not by Ponzo (1900), is documented by a single specimen collected by Gasparrini and preserved in PAL. Furthermore, extensive field investigations conducted in recent years on the nearby and ecologically similar cliffs of the adjacent mountains (namely Mt. Monaco, Mt. Palatimone and Mt. Passo del Lupo) were unsuccessful (LDS, L. Scuderi, SP, pers. obs.).

The occurrence of *E.sicula* in Malta was communicated by G. Gulia to C. F. Nyman during his visit to the island in 1844. In his subsequent report on Malta’s vegetation, Nyman (1846) only cited *Ericamultiflora* L., whilst the first official mention of *E.sicula* came from Duthie (1875) and was later cited by several other authors (Nyman 1879; Caruel 1889; Fiori and Paoletti 1898). However, Sommier and Caruana-Gatto (1915) pointed out the absence of herbarium specimen, and more recently, this record has been considered a misidentification, leading to the exclusion of the species from the Maltese vascular flora (Brullo 1982; Lanfranco 1982).

#### ﻿Lebanon

**Figure 4.**
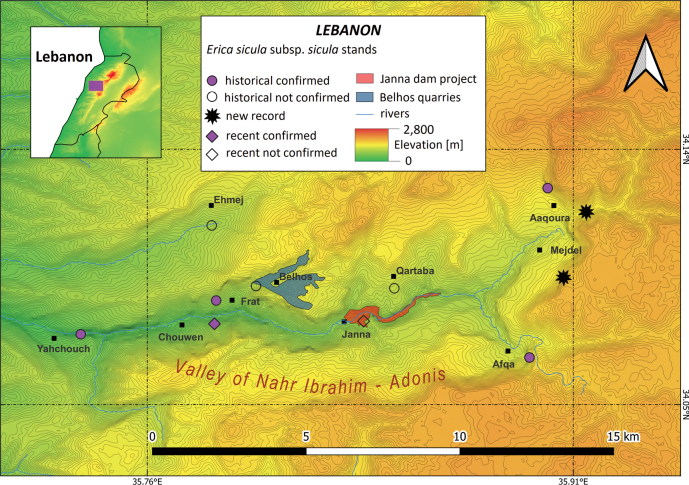
Past and present distribution of Ericasiculasubsp.sicula in Lebanon based on literature data and herbarium specimens and on field data collected between 2022 and 2023 by one of the authors (HEZ). Belhos quarries and Janna dam correspond to the areas where the local stands were recently wiped out due to habitat destruction.

In Lebanon, E.siculasubsp.sicula occurs in the Nahr Ibrahim Valley, historically known as the River of Adonis, located in the central part of Mount Lebanon. This taxon shows a scattered distribution as it occurs in two separated areas in the lower and the upper part of the valley (Fig. 4). The first is a canyon known as the Gorges of Yahchouch, the other includes several steep cliffs overlooking Aqoura and Afqa.

In the following lines we present a diachronic overview of the finds of E.siculasubsp.sicula in Lebanon. The taxon was first collected at Belhos in 1880 (also known as Billa, Billaa, Bil’âs, or Balhas), below the bridge between Machnaqa and Qartaba, by L.C.É. Lortet. The specimen was subsequently given to W. Barbey, who made the first description of the taxon Pentaperasiculavar.libanotica (Barbey-Boissier and Barbey 1882). The plant was later collected in the surroun­ding areas: at Ehmej (also Ihmish and Ehmège) by T. Boutros in 1890 (Mouterde 1983), Qartaba (also Kartaba) by E. Hartmann in 1898 (Browicz 1983), at Frat and at Milassa by G. Post in 1908 (Post and Dinsmore 1932). The taxon was also collected in the gorges of Yahchouch and at Aaqoura (also Aakoura and Akoura) by R. Gombault, J. Louis and J. Thiébaut in 1932 (Mouterde 1983). It was collected for the first time at Afqa (also Afka) under the river catchment, by P. Mouterde in 1935 (Mouterde 1983). H.A. Pabot also collected a specimen in the gorges of Yahchouch in 1953 (Mouterde 1983). E.siculasubsp.sicula was recently observed at Chouwen, which is located within the buffer zone of the Jabal Moussa Biosphere Reserve (Tohmé and Tohmé 2014) and at Janna (Stephan et al. 2017).

Our field surveys enabled the mapping of seven extant stands. The locality of Milassa could not be located and the specimen remains untraceable. Actually, there is no village named Milassa in Lebanon. The most similar toponym is Lassa, but we chose to exclude this unplaced occurrence recorded by Post. We confirmed the destruction of two stands: the stand once located in Belhos was destroyed approximately 20 years ago by quarrying activities (Stephan et al. 2017), which resulted in the loss of about 302 ha of suitable habitat. The second stand, formerly located on the cliffs of the canyon in Janna, was destroyed by the construction of a dam that began in 2014. The entire locality, covering an area of 85.66 ha, was excavated and the adjacent cliffs were stripped up to a height of 50 metres. Despite extensive and repeated surveys in the area, the nucleus of Ehmej could not be located. This area, intensely exploited by stone quarries, has experienced significant degradation of its cliff habitats. The specimen labelled as collected in Qartaba was probably collected on the road between Qartaba and Janna, as no nucleus was observed in Qartaba. The stands of Yahchouch, Chouwen and Frat were confirmed in the lower part of the canyon. The stand of Afqa was confirmed, found just above the cave where the river of Ibrahim-Adonis begins its flow. Two additional stands were found in the upper part of the valley, in Mejdel and East Aqoura. The four nuclei of the upper valley, namely Afqa, Mejdel, Aqoura and East Aqoura are the largest in terms of size.

#### ﻿Cyprus

**Figure 5.**
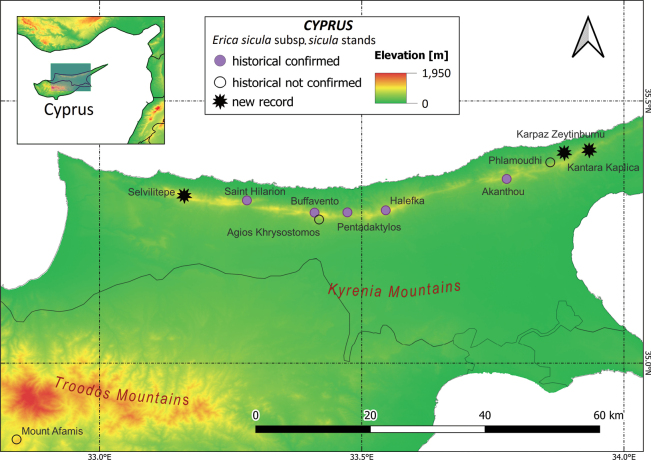
Past and present distribution of Ericasiculasubsp.sicula in Cyprus Island based on literature data and herbarium specimens (Meikle 1985) and on the occurrence data collected during the field surveys carried out in 2022 by one of the authors (SG).

Eight stands of E.siculasubsp.sicula occur in Cyprus, all within the Kyrenia Mountain range (Fig. 5). Meikle (1985) provides a detailed list of historical records: the taxon was first found by Kotschy at Pentadaktylos in April 1859 and at Agios Khrysostomos in 1862 (Unger and Kotschy 1865). The nucleus of Buffavento, first noted by Kotschy (1862), has been repeatedly observed in the recent years, including by Syngrassides in 1937 (Meikle 1985), by S. Brullo and G. Giusso del Galdo in September 2013 (Wagensommer 2017), and by S. Cambria in June 2019 (pers. comm.). Other early records refer to a nucleus near Akanthou (‘Sintenis and Rigo Iter Cyprium 1880’, ‘Merton 1967’ in Meikle 1985) and in the gorges west of Saint Hilarion (‘Hartmann 1904–1905’, ‘Casey 1951’ in Meikle 1985); recent surveys confirm the species persists at both sites (SG, pers. obs.). Additional stands were recorded between the 1930s and 1960s: at Yaïlá (district Alevkaya-Halevga) by Kennedy and Davis between 1938 and 1941, at Halevga by Davis between 1940 and 1949, and above Phamoudhi (district of Kyrenia) by Merton in 1967. In 2006, a new stand was found at Karpaz Zeytinburnu and another close to a historical collection site at Alevkaya on the road to Esentepe. Two additional new sites were discovered in 2020 on the highest peaks of the Kyrenia Mountains at 1,024 m a.s.l. at Kyparissovouno (= Selvilitepe, authors’ note) and at Kantara Kaplica.

The only historical record from the Troodos Mountain range is from Mount Afamis (c. 1,050 m a.s.l.) in the Limassol-Lemesos District, reported by Kennedy in 1937 (Meikle 1985). However, this stand has not been confirmed despite repeated *ad hoc* expeditions carried out between 2003 and 2007.

#### ﻿Türkiye

**Figure 6.**
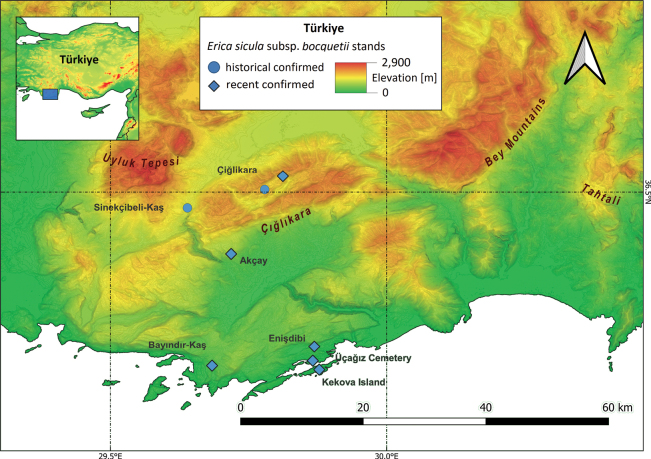
Current distribution of Ericasiculasubsp.bocquetii in Türkiye based on literature data and herbarium specimens and on recently recorded occurrence data.

E.siculasubsp.bocquetii, locally known as ‘yılgun çalısı’ (OŞ, pers. comm.), was discovered and described by Peşmen (1968). Its type locality is located at about 1,750 m a.s.l. in a clearing of *Cedruslibani* A. Rich. forest at Çiğlikara, Dokuzgöl Mevkii, in western Taurus (SW Anatolia, province of Antalya, district of Elmalı; see Fig. 6). A specimen in the herbarium of J. Bornmüller at Berlin suggests this taxon was collected in the same area in 1938. More recently, it has been observed growing under similar ecological conditions and repeatedly collected between 1,600 and 1,850 m a.s.l., close to the type locality, within the Elmalı Cedar Research Forest (Yaltırık 1971; McClintock 1990, 1991; Kendir 2005; Kendir and Güvenç 2008; Ari et al. 2014).

Another stand was found by Burton (1995) at approximately 1,200 m a.s.l. growing under warmer climatic conditions on the steep limestone outcrops of an area dominated by *Pinusbrutia* Ten., located between Sinekçibeli and Kaş (province of Antalya).

During the last 15 years E.siculasubsp.bocquetii, previously thought to grow exclusively in mountainous areas, has been found at much lower altitudes; in fact, a stand located at 250 m a.s.l. was discovered in 2009 near Bayındır-Kaş (Z. Aytaç, pers. comm.). Ten years later, Dilek (2018 and 2020) reported three more stands, located near Enişdibi and close to Üçağız Cemetery (both near Demre, Antalya) and on Kekova Island, whilst Fener (2018) and Fener and Aykurt (2019) reported its occurrence at 762 m a.s.l. at Çığlıkara Püreni, and there is an herbarium specimen (“Gülkokan 1871”) collected in the roadsides between Akçay and Kemer.

**Figure 7.**
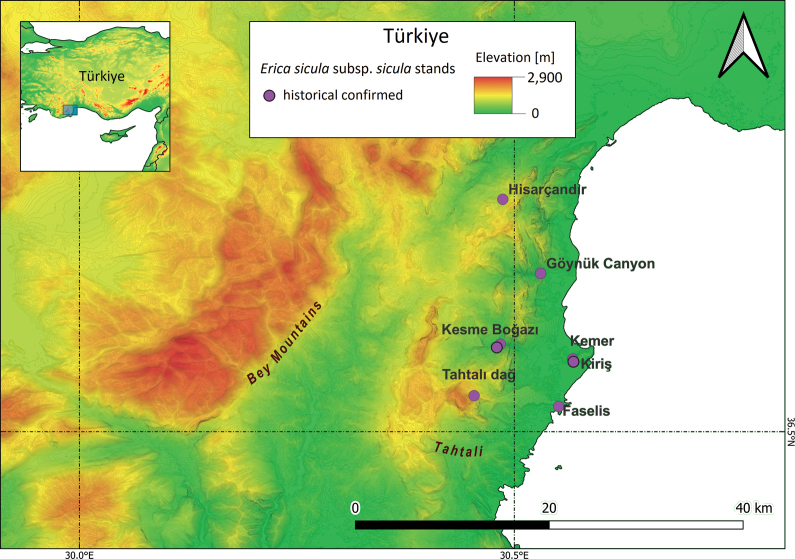
Current distribution of Ericasiculasubsp.sicula in Türkiye based on literature data and herbarium specimens and on recently recorded occurrence data.

E.siculasubsp.sicula has been reported from several localities in the southeastern sector of the Antalya province in southwestern Anatolia (Fig. 7). Eight collection sites have been documented. Specifically, it was first observed at Kezme Boğazi, south of Kemer, in 1947 and at Göynük Canyon (Stevens 1978; Browicz 1983). A third stand was observed in 1960 at Tahtalı Dağ, followed by Hisarçandır-Karlıktepe in 1978, around Phaselis Bay in 1979, around the coast of Kemer in 1980, and above the ruins of Phaselis in 1995. More recently, a stand was found by Gülkokan et al. in 2015 (Fener 2018; Fener and Aykurt 2019; Gülkokan is the surname of D. Fener prior to her marriage, Authors’ note) on the calcareous stony slopes near Kemerköy, although the exact collection site could not be localised. Finally, recent field surveys led to the discovery of two stands very close to the historical collection sites of Kesme Boğazı and Phaselis Bay.

#### ﻿Libya

**Figure 8.**
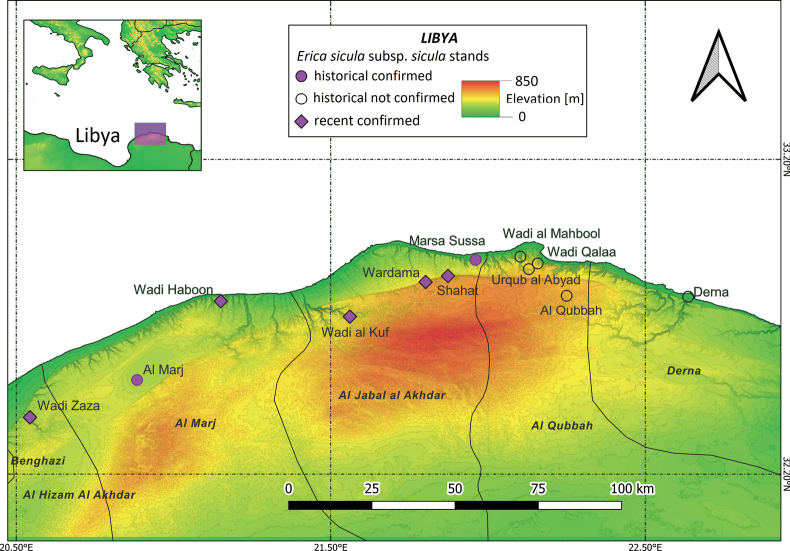
Distribution of Ericasiculasubsp.sicula in Libya based on literature data and herbarium specimens.

E.siculasubsp.sicula, locally known as ‘hamra’ (Siddiqi 1978), occurs in the hilly regions of N Cyrenaica subject to Mediterranean climatic conditions, namely in the districts of Al Hizam Al Akhdar, Al Marj, Jabal Al Akhdar, Al Qubbah and Derna. The distribution map (Fig. 8) of the Libyan population was based on literature data (e.g., Durand and Baratte 1910; Béguinot and Vaccari 1913a, 1913b, 1913c; Pampanini 1931; Brullo and Furnari 1979, 1994; Siddiqi 1978) and herbarium specimens. It shows the location of seven stands consi­dered still extant, and of five historical collection sites not confirmed since the 1970s. E.siculasubsp.sicula was first observed near the city of Derna/Darnah (‘Haimann 1881’ and ‘Taubert 1887’ in Durand and Baratte 1910), at Wadi Sarak (‘quite common in the woods’) and Wadi Naga (‘mountains at the north of the valley’: ‘Taubert 1887’ in Durand and Baratte 1910). Both Haimann and Taubert sent numerous specimens to European herbaria, quickly disseminating their discovery to the scientific community (e.g., Hooker 1888). In the follo­wing decades, additional stands were sampled at Barca (also Barqa or Barce, today’s Al Marj: Arcangeli 1894), Gouba (also El Gubba, Quba, Al Qubbah) and Wadi Derna (‘Vaccari 1912’ in Béguinot and Vaccari 1913a, ‘Longa 1912’ in Pampanini 1912), Marsa Susa (ancient Apollonia, Authors’ note; ‘Vaccari 1913’ in Béguinot and Vaccari 1913b), and ‘in the valley of the aqueduct’ (‘Vaccari 1913’ in Béguinot and Vaccari 1913c). More recently, E.siculasubsp.sicula was observed at Wadi Zaza (El-Barasi et al. 2003), Wadi Haboon (El Rabiai and Al Tira 2015), whilst Omar (2019a) reported its occurrence in the maquis at Marsa Susa (180–195 m a.s.l.), the forest at Wardamah (500–550 m a.s.l.) and at Shahhat (500–550 m a.s.l.).

### ﻿Ecology of *Ericasiculas.l.*

#### ﻿Italy

In NW Sicily, E.siculasubsp.sicula grows on dolomitic cliffs and ledges between 100 and 630 m a.s.l. and may be considered a primary chasmophyte. This taxon is a characteristic species of a plant community rich in endemic taxa only occurring on Mt. Cofano, described as the phytosociological subassociation *Scabioso limonifoliae-Centauretum ucriae* subass. *ericetosum siculae* (Brullo and Marcenò 1979; Gianguzzi and La Mantia 2008). Near the ledges, it may co-occur with other woody species, such as *Quercusilex* L., *Arbutusunedo* L., *Pistacialentiscus* L., *P.terebinthus* L. and *Ericamultiflora* L. or with the tussock grass *Ampelodesmosmauritanicus* (Poir.) Dur. & Schinz. Field surveys by de Simone (2020) found E.siculasubsp.sicula absent on S-facing slopes, but present on all other orientations. A plot-based analysis showed that it has an ecological preference for east- and west-facing cliffs with inclinations between 60° and 90°. Moreover, it prefers ridges exposed to cool and humid breezes rather than cliff bases (de Simone 2020).

#### ﻿Lebanon

E.siculasubsp.sicula typically grows between 400 and 1600 m a.s.l. (Mouterde 1983; Browicz 1983; Mugrabi de Kuppler 2013), preferring N-facing rocky slopes and cool, shady microclimates at the bottom of gullies (Gombault 1946; Bou Dagher-Kharrat et al. 2013). According to the zonation of Abi-Saleh (1982), it ranges within the meso- to supra-Mediterranean levels. Field surveys repor­ted it growing in at least five habitat types, co-occurring with diverse species. In the lower Ibrahim Valley, on both N- and S-facing slopes, near Yahchouch, Chouwen and Frat, it occurs in meso-Mediterranean *Quercuscoccifera* woodlands and *Pinusbrutia* woodlands (habitat types T213_LB1 and T3A5 accor­ding to El Zein et al. 2022). Typically accompanying species are *Pinusbrutia*, *Arbutusandrachne* L., *Ptilostemonchamaepeuce* (L.) Less, Rosulariasempervivumsubsp.libanotica (Labill.) Eggli, *Sedum* spp., and Dryopterispallida(Bory)Maire et Petitm.subsp.libanotica (Rosenst.) E. Nardi (Stephan et al. 2017). In the upper Ibrahim Valley, the plant occurs on N- and W-facing cliffs between 1,400 and 1,600 m a.s.l., in scattered supra-Mediterranean *Quercuscoccifera* L. woodlands (habitat T213_LB2), *Ostryacarpinifolia* Scop. woodlands (habitat T19B1_LB1) and *Juniperusdeltoides* R.P. Adams thickets (habitat S2314), with scattered individuals of *Pistaciaterebinthus* L., *Potentillalibanotica* Boiss., *Onosmafrutescens* Lam. and *Hirtellinalobelii* DC.

#### ﻿Cyprus

E.siculasubsp.sicula occurs between 275 and 975 m a.s.l., mostly growing on N-facing sites, but on S-facing slopes at its highest location at Buffavento. It typically thrives in the crevices of hardly accessible cliffs, occasionally in sclerophyllous maquis and within gaps among natural or artificial conifer woodlands of *Pinusbrutia* Ten. and *Cupressussempervirens* L. on mountain slopes (Meikle 1985; Viney 1994; Hand et al. 2011; Wagensommer 2017).

#### ﻿Türkiye

According to Stevens (1978), E.siculasubsp.bocquetii grows on N-facing limestone cliffs within *Cedruslibani* woodlands in the montane-Mediterranean vegetation belt in Çığlıkara Nature Reserve. More recently this taxon has been recorded also under fully thermo- and meso-Mediterranean conditions, always on N-facing limestone cliffs, ledges and cracks.

The Turkish stands of E.siculasubsp.sicula are located near the coast between 60 and 100 m a.s.l., under fully thermo-Mediterranean climatic conditions, often accompanied by *Globulariadavisiana* O. Schwarz (Stevens 1978).

#### ﻿Libya

In Cyrenaica E.siculasubsp.sicula grows on the cliffs and ledges of steep calcareous slopes along seasonal streamsides (called ‘widien’, plural of ‘ouadi/wadi’), between (100)250 and 500 m a.s.l. (Browicz 1983; Brullo and Furnari 1994). According to Brullo and Furnari (1994), it participates in various chamaephytic communities, including garrigue formations such as the *Asperulo tragacanthoidis-Rosmarinetum offinalis* (Susa and el-Hilal, 180–310 m a.s.l.), and chasmophilous assemblages like the *Origano cyrenaici-Putorietum calabricae* in a wadi beneath Susa and el-Hilal (100–310 m a.s.l.), and the *Telephio barbeyani-Darnielletum cyrenaicae* in Wadi Derna (260–300 m a.s.l).

### ﻿Biology of *Ericasiculas.l.*

Concerning the vegetative growth, Raunkiær (1934) probably observed the behaviour of an individual of *Pentaperasicula* cultivated in the Botanic Garden of Copenhagen, noting that the plant was able to produce dwarfed and denser foliage on shorter shoots under stressful conditions. Vegetative propagation trials with woody cuttings of E.siculasubsp.sicula from Türkiye, harvested in February, showed low rooting success (43.5%). Success was even lower (<20%) during harvesting periods and for subsp. bocquetii (Ari et al. 2015). Green cuttings from NW Sicily also showed low success, suggesting the need for improved techniques (A. Cristaudo, pers. comm.).

Information on reproductive biology (e.g., breeding system, seed duration and seedling survival) is scarce. Accessions collected for the Genmedoc and Semclimed Projects in the 2000s and stored in the germplasm bank of the Department of Biological, Geological and Environmental Sciences of the University of Catania (BGS-CT) contained no viable seeds. This fact may be due to low reproductive performance, particularly in plants growing on the foothills of Mt. Cofano (A. Cristaudo, pers. comm.).

Germination tests on Lebanese E.siculasubsp.libanotica (now considered part of E.siculasubsp.sicula) at the University Saint Joseph of Beirut showed that seeds are orthodox, with a germination rate of approximately 25% after 30 days at 16 °C on agar with alternating 12 h light/12 h dark cycles (Lebanon Flora 2020). In contrast, another Lebanese accession studied at Kew achieved 100% germination after 63 days at 10 °C on 1% agar under an 8 h light/16 dark regime (source: SER INSR-RBGK 2023; https://ser-sid.org/species/5dc3038b-0eb7-4a23-b665-3cec9a4aacc8 and https://ser-sid.org/species/1aed0b53-1f32-4a6f-9df8-c9036134337c).

Both subspecies of *Ericasicula* possess pollen tetrads, as verified in Lebanese (Haddad 1969) and Cypriot (Sarwar 2007) stands of E.siculasubsp.sicula, as well as in E.siculasubsp.bocquetii (Pınar and Oybak 1995).

Moreover, neither the pollination ecology nor the dispersal strategies of the species complex have been properly investigated yet. Within the genus *Erica* pollination is often by insects (Rebelo et al. 1985; Ballantyne et al. 2015; Bouman et al. 2017; Moquet et al. 2017; Heystek and Pauw 2014). The seeds of the pentamerous heathers are very light 0.5–0.9 mg), small (0.5–0.8 × 0.3–0.5 mm) and winged (Fagúndez and Izco 2011), suggesting wind dispersal, although the distance they might travel before landing on suitable habitat remains unknown.

### ﻿Census and measure of AOO and EOO at the global and national scale

The overall EOO of *Ericasiculas.l.* (i.e., including both subspecies) exceeds 715,000 km^2^, whilst the AOO is only 140 km^2^. Fig. 2 displays the extent of the species’ range throughout the Mediterranean Basin.

#### ﻿Italy

**Figure 9.**
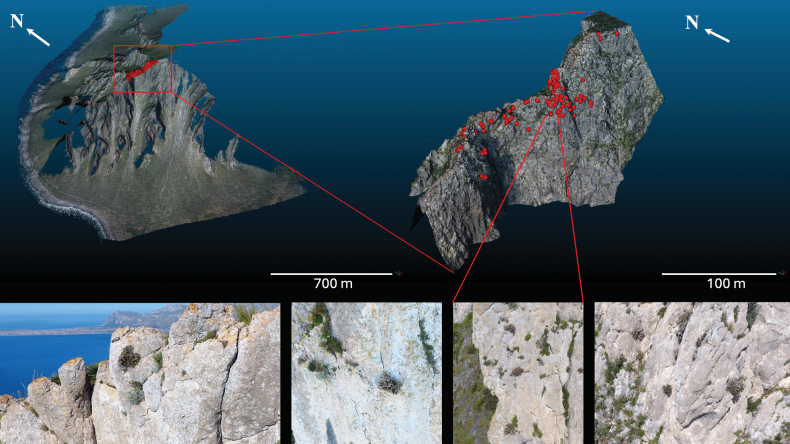
Results of UAS-based monitoring of the Italian subpopulation. The red surface in the upper left image points out a target site hosting E.siculasubsp.sicula located on Mt. Cofano (Sicily); the red points in the point cloud of the upper right image correspond to the individuals growing on the cliff, whose identity and exact position was recorded through the photos taken by the drone (see image sequence below).

A UAS census at Mt. Cofano precisely located 985 individuals (Fig. 9) in four sampled areas in suitable habitat, covering a total cliff surface of 5.91 ha (de Simone 2020). Considering the cliff inclination within the grid cells where the taxon was confirmed, and using a 2 × 2 m digital model, we calculated a real AOO of 57.84 ha, while the projected AOO was only 25.12 ha. Consequently, when using the minimum convex polygon method, the EOO was found to be 100.63 ha.

Assuming the surveyed areas represent the total stand and considering the total extent of the cliffs where the species was observed, the total subpopulation in Sicily is estimated between 4,186 and 9,638 individuals, with the oldest and largest ones (crown > 2 m) growing at higher altitudes (de Simone 2020). However, this may be an overestimate of the actual subpopulation size, as the cliffs are not homogeneously colonized due to micro-topographic preferences (de Simone 2020). Indeed, the assumption of homogeneous occupation may underestimate the impact of local abiotic (e.g., slope, rockiness) and biotic (e.g., competition with other rupicolous species) factors.

#### ﻿Lebanon

Fieldwork carried out between 2022 and 2023 enabled a more precise calculation of the EOO and AOO of E.siculasubsp.sicula, whose values are 55.067 km^2^ and 28 km^2^, respectively.

#### ﻿Cyprus

Recent surveys have provided detailed information about the 8 subpopulations inhabiting the island. Based on 2 × 2 km wide cells, the EOO of the Cypriot subpopulations is around 327.1 km^2^, with an AOO of 32 km^2^.

#### ﻿Türkiye

For the 8 subpopulations for E.siculasubsp.bocquetii, we estimated an EOO of 595.6 km^2^ and an AOO of 32 km^2^.

Based on the location of the 8 subpopulations of E.siculasubsp.sicula in Antalya province, the EOO of the Turkish subpopulations is about 153.6 km^2^, with an AOO of 24 km^2^.

#### ﻿Libya

Based on the geographic coordinates of the herbarium specimens, we were able to map most of the historical records of E.siculasubsp.sicula in Libya. Seven stands were confirmed, which allowed us to estimate an EOO of 1,464 km^2^ and an AOO of 28 km^2^.

### ﻿Current conservation, main threats and demographic trends

The minimum viable value for *E.sicula* was estimated to be 500–1000 mature individuals per subpopulation (Commission of the European Communities 2009).

#### ﻿Italy

Most likely, two out of three Sicilian stands have disappeared during the last two centuries. The whole population falls within the Special Area of Conservation of the Natura 2000 network ITA010016 “Monte Cofano e littorale” and in the regional nature reserve ‘Monte Cofano”. Mt. Cofano is also an Important Plant Area (hereinafter IPA; Blasi et al. 2010). *E.sicula* is featured in all the Italian (Conti et al. 1992; Conti et al. 1997; Scoppola and Spampinato 2005; Rossi et al. 2013) and Sicilian (Raimondo et al. 1994; Raimondo et al. 2011) red lists. The increasingly frequent wildfires which recently affected Mt. Cofano represent the only factor currently affecting this local subpopulation. Another threat is the oversampling of plant specimens. In fact, more than 80 specimens collected in the *locus classicus* of Mt. Cofano are found in European herbaria (see Suppl. material 1), suggesting that, even if most plants are safe from collectors, the more accessible portion of the local subpopulation has been systematically damaged for two centuries by botanists eager to possess their own specimen. Additionally, the fast spread in the adjacent municipalities of the invasive alien perennial grass *Cenchrussetaceus* (Forssk.) Morrone (Scuderi and Pasta 2009), may soon pose a serious threat to the Italian subpopulation.

#### ﻿Lebanon

E.siculasubsp.sicula is severely threatened by the increasing impact of human activities. In fact, the primary threats to most of the Lebanese subpopulations are quarrying, urban sprawl and the construction of built infrastructure such as dams and roads. The increasingly frequent fires severely affect some of the stands, especially in the lower parts of the canyon and constitute a secondary threat. Overgrazing occurs in the upper part of the valley and may hinder the expansion of the few remnant nuclei in cliff areas accessible to goats. The total subpopulation size in Lebanon is estimated at 5,000 individuals, with 4,000 distributed in the areas of Afqa, el Mejdel and Aaqoura, and 1,000 in the area of Yahchouch Chouwen and Frat. At least two of the known subpopulations were destroyed in the two last decades by quarrying and the construction of the dam of Janna. Only a small part (<10%) of the subpopulations fall into the territory of Jabal Moussa Biosphere Reserve. Although the entire Nahr Ibrahim Valley has been identified as an IPA according to two studies (Radford et al. 2011; Bou Dagher-Kharrat et al. 2018) and as a Key Biodiversity Area (El Zein et al. 2018), only the small area included in Jabal Moussa is a protected area. Hence a total of 7 location were identified, 6 of them being currently threatened.

#### ﻿Cyprus

E.siculasubsp.sicula grows in areas of high conservation interest, but these sites currently are not yet protected by law. The most recent field census suggests an overall number of approximately 4,700 individuals forming seven distinct subpopulations, whose number and size are expected to shrink in the long term due to goat overgrazing and to increasingly frequent wildfires (SG, pers. comm.). Although some of its stands became extinct during the last decades, this taxon is not included in the Red Data Book of the Flora of Cyprus (Tsintides et al. 2007).

#### ﻿Türkiye

In the IUCN Red Data Book of the Turkish Vascular Flora (Ekim et al. 2000), both E.siculasubsp.bocquetii and E.siculasubsp.sicula(undersubsp.libanotica, Authors’ note) were classified as Vulnerable (VU), while Tezel et al. (2020) cite E.siculasubsp.bocquetii as Critically Endangered (CR) with no further details.

Moreover, the type locality of E.siculasubsp.bocquetii falls within the Çığlıkara Tabiat Koruma Alanı (= Çığlıkara Nature Reserve) (OŞ, *pers. obs*.), while some of the Turkish subpopulations of E.siculasubsp.sicula fall within the Beydağları Sahil Milli Parkı (= Beydağları Coastal National Park) and/or the Kaş-Kekova Special Environmental Protection Area.

#### ﻿Libya

The lack of recent updates concerning this country does not allow us to eva­luate the exact number and the size of local subpopulations. All of them grow in the Jabal Al Akhdar Region, a territory that has been designated as IPA by El-Rtaib (2010) and by Radford et al. (2011), but that is not protected by law. Threats to vegetation in the massif of Jabal Al Akhdar are well documented. Human activities were relatively more restricted in this area in the past, aimed at protecting the forests. However, the situation has changed during the last decade following the political instability, which led to civil unrest and movement of human populations. The main threats are firewood collection, charcoal production, over-harvesting of medicinal and aromatic plants, bush fires, quarrying, urban sprawl, overgrazing, agriculture expansion and uncontrolled camping (Saaed et al. 2022). The habitat extent and quality are still declining now. Several dams were built along Wadi Derna in the 1970s to prevent seasonal floods and to collect water for irrigation purposes. These dams sadly hit the headlines in September 2023 because of their collapse after an intense rainstorm, causing thousands of human casualties. Many Libyan subpopulations of E.siculasubsp.sicula, which were concentrated within the catchment area of this seasonal stream might have been severely affected by this event.

## ﻿Discussion

### ﻿Ecology

Both subspecies *Ericasicula* group grow exclusively on vertical cliffs (cha­smophytes) or in ledges and large fractures (comophytes). Such ecological behaviour proves to be a globally rare feature among heathers. Additionally, they grow only on limestones and dolomias, whereas most Ericaceae thrive on acid or very acid soils (Webb and Rix 1972; Oliver 1991). Although these very unusual edaphic requirements are intriguing, there is currently no available information on the soil biota (e.g., ericoid mycorrhizae capable of capturing phosphorus and nitrogen) connected with *Ericasiculas.l.*

Although they all share the same habitat, the different populations of E.siculasubsp.sicula differ in terms of altitudinal range strikingly enough to raise taxo­nomic questions. For instance, the Italian, Turkish and Libyan populations of Ericasiculasubsp.sicula only occur under strictly thermo-Mediterranean climate, whereas the Cypriot and Lebanese populations grow only on mountain ranges subject to meso- and supra-Mediterranean climatic conditions.

The remarkably wide altitudinal range of E.siculasubsp.bocquetii probably induces striking differences in the blossoming times between its lowland and the mountain subpopulations (June to September), thus causing significant reproductive isolation between them (McClintock 1980). Moreover, the finding of E.siculasubsp.bocquetii at lower altitudes makes it necessary to verify its biological distinction from subsp. libanotica, with which it is sympatric.

### ﻿Extinction risk assessment

Table 4 provides a synthetic overview of the thresholds, and the criteria followed to perform the extinction risk assessment both at the global/species scale and at the subspecies-regional scale.

**Table 4.** Overview of the thresholds and criteria followed for the extinction risk assessment of the taxa of the *Ericasicula* group at the species-global scale and at the subspecies-national scale; n.a. = not assessed. AOO = Area of Occupancy; EOO = Extent of Occurrence.

Taxon	Country	Nb. of Stands	EOO (km^2^)	AOO (km^2^)	Estimated subpopulation size (nr)	Severely fragmented (Y/N)	Threats	Criteria	Status
E.siculasubsp.sicula	Italy	1	4	4	4,186-9,638	N	wildfires, invasive alien plants	B1ab(i,ii,iii,iv) + 2ab(i,ii,ii,iv)	CR
E.siculasubsp.sicula	Libya	7	1,464	28	n.a.	N	firewood collection for charcoal production, overharvesting for medicinal purposes, wildfires, quarrying, urban sprawl, overgrazing, dam construction	B1ab(iii) + 2ab(iii)	VU
E.siculasubsp.sicula	Cyprus	8	327.1	32	4,700	N	overgrazing, wildfires	B1ab(i,ii,iii,iv) + 2ab(i,ii,ii,iv)	VU
E.siculasubsp.sicula	Türkiye	8	153.6	24	n.a.	N	urban sprawl	B1ab(i,ii,iii,iv) + 2ab(i,ii,ii,iv)	VU
E.siculasubsp.sicula	Lebanon	7	55.1	28	5,000	N	quarrying, dam construction, road construction, urban sprawl, wildfires	B1ab(i,ii,iii,iv) + 2ab(i,ii,ii,iv)	VU
E.siculasubsp.bocquetii	Türkiye	8	595.6	32	n.a.	N	overgrazing, wildfires	B1ab(iii) + 2ab(iii)	VU
*E.sicula* *sensu lato*	Global	39	715,366	140	n.a.	Y		B2ab(i,ii,iii,iv)	LC

At the global/species scale *E.siculasensu lato* was evaluated as of Least Concern (LC). On the one hand, the EOO is rather extensive, with 715,366 km^2^; on the other hand, the AOO is of 140 km^2^ and a continuing decline was observed (b) in EOO (i), AOO (ii), area, extent and/or quality of habitat (iii) and number of subpopulations or locations (iv). Although each subpopulation is capable of surviving within the five countries, the global distribution pattern is severely fragmented. Considering the considerable distances between *E.sicula* subpopulations, only those in Cyprus may be interconnected with those of Lebanon and Türkiye. However, the potential for long-distance dispersal by pollinators allowing effective gene flow, as well as the requirements for seed dispersal, remain unproven. It is likely that no subpopulation is sufficiently close to allow regular gene flow, except under exceptional circumstances, which raises concerns about genetic depletion as a significant threat. The situation of Ericasiculasubsp.sicula is worrying, because it has undergone substantial population decline over the past fifty years across its entire distribution range. In fact, two of the three known stands in northwest Sicily (Italy) are now definitively extinct; in Lebanon, two new stands were discovered, along with five confirmed and four unconfirmed, including two that have been proven extinct. Similarly, Cyprus has three new stands, five confirmed and three unconfirmed, while in Türkiye, there are two new, two confirmed and five extinct stands.

#### ﻿Italy

Based on 2 × 2 km wide cells, Domina et al. (2012) previously estimated the EOO and AOO at 4 km^2^ each, considering the surface effectively occupied by E.siculasubsp.sicula around 25 ha. These authors listed the species as Critically Endangered (CR) based on the subcriterion B1ab(i,ii,iv,v). Based on the data collected during the fieldwork by de Simone (2020), we are able to confirm the risk level assigned to this subspecies; yet, we applied different and additional sub-criteria - B1ab(i,ii,iii,iv) + 2ab(i,ii,iii,iv) - to support the previous assessment: the EOO (4 km^2^) and AOO (4 km^2^) are very restricted (B1 + B2), there is one sole location (a) and a continuing decline observed (b) in EOO (i), AOO (ii), area, extent and/or quality of habitat (iii), number of subpopulations or locations (iv).

#### ﻿Lebanon

The discovery of additional subpopulations led to larger values for EOO and AOO compared to the previous assessment (Stephan et al. 2017), which were 45 km^2^ and 20 km^2^, respectively.

The updated data collected during surveys on distribution, number of subpopu­lations and threats have allowed us to review the EN status previously evaluated in the initial assessment (Stephan et al. 2017). We assessed the taxon as VU at the national scale of Lebanon under the subcriteria B1ab(i,ii,iii,iv) + 2ab(i,ii,iii,iv): the EOO (55.067 km^2^) and AOO (28 km^2^) are very restricted (B1 + B2), there are seven locations (a) and a continuing decline observed (b) in EOO (i), AOO (ii), area, extent and/or quality of habitat (iii), number of subpopulations or locations (iv).

#### ﻿Cyprus

The data yielded during the recent field investigations allowed us to assess E.siculasubsp.sicula as VU at the island scale under the subcriteria B1ab(i,ii,iii,iv) + 2ab(i,ii,iii,iv): both the EOO (327.1 km^2^) and the AOO (32 km^2^) are very restricted (B1 + B2), there are eight locations (a) and a continuing decline observed (b) in EOO (i), AOO (ii), area, extent and/or quality of habitat (iii), number of subpopulations or locations (iv).

#### ﻿Türkiye

E.siculasubsp.bocquetii was previously evaluated as Critically Endangered (CR) according to the criteria B1a+2a, as already suggested by AKS Planlama Mühendislik Ltd Şti. (2010). Following updated analyses, we assessed the taxon as VU at the global scale under the subcriteria B1ab(iii) + 2ab(iii): EOO (595.6 km^2^) and AOO (32 km^2^) are restricted (B1 + B2), there are eight locations (a) and a constant decline (b) in the (iii) area, extent and/or quality of habitat was observed.

E.siculasubsp.sicula was evaluated as nationally VU under the subcriteria B1ab(i,ii,iii,iv) + 2ab(i,ii,iii,iv): the EOO (153.6 km^2^) and AOO (24 km^2^) are very restricted (B1 + B2), there are six locations (a) for eight stands, and a continuing decline observed (b) in EOO (i), AOO (ii), area, extent and/or quality of habitat (iii), number of subpopulations or locations (iv).

#### ﻿Libya

E.siculasubsp.sicula was assessed as VU at the national scale under the subcriteria B1ab(iii) + 2ab(iii): the EOO (1,464 km^2^) and the AOO (28 km^2^) are restricted (B1 + B2), there are seven locations (a) and a continuing decline observed (b) in the area, extent and/or quality of habitat observed (iii). It is worth underlining that the Libyan stands are currently affected by many severe and synergic threats (Alawamy et al. 2020).

## ﻿Conclusions

The information provided in this paper may be used to enhance the protection of all known and threatened subpopulations of the taxa referred to *Ericasiculasensu lato.* Identifying knowledge gaps is a crucial step for effective plant conservation. By pinpointing missing information, efforts can be directed to address those gaps in order improve our understanding of ecological requirements of the target species and to develop effective mid- and long-term protection measures.

The global conservation status of a taxon is influenced by the conditions and challenges it faces in the countries where it occurs. However, as illustrated by the case of *Ericasicula*, the global status may not accurately reflect the distinct risks of extinction in each individual country. This observation could also underscore a potential flaw in the guidelines and criteria of the IUCN Red List. Consequently, it is essential to carefully consider the regional assessments when evaluating species with many subspecies and subpopulations scattered across extensive ranges. To accurately understand and mitigate species extinction risk, a careful examination of the status and threats faced by each subspecies is required.

All taxa within this group inhabit very steep slopes, exhibit extremely low reproductive performance, have narrow ranges and show fragmented and declining population at the national scale. These characteristics, coupled with low competitivity and ongoing population shrinkage, are probably triggered by multiple factors. Therefore, future investigations should adopt a multifaceted approach.

For instance, we need to record detailed diachronic data on the microclimatic requirements of the pentamerous taxa using data-loggers (Marcenò et al. 2022). In fact, these heathers likely benefit from favourable climatic conditions, such as cool summers at higher altitudes, and microclimatic factors, such as overnight moisture near the coasts or riverbeds, which help reduce seaso­nal water stress. Notably, de Simone (2020) recorded consistent air humidity and daily temperature values in the north-facing cliffs of Mt. Cofano (Sicily). This finding helps explain why attempts to plant E.siculasubsp.sicula at lower altitudes on Cyprus Island were unsuccessful (SG, pers. comm.). The use of drones to monitor the subpopulations can facilitate better understanding of the auto- and synecology of these cliff-dwelling taxa in otherwise inaccessible contexts (de Simone 2020; Reckling et al. 2021; Li et al. 2022). When combined with detailed climatic data, occurrence records and ecological data, these efforts can significantly improve the quality of IUCN risk assessments and the precision of niche models (e.g., Bazan et al. 2012).

Intensive prospections and increased sampling effort are needed, particularly for the less investigated subpopulations of Türkiye and Libya, to assess the genetic diversity within and among all members of the *Ericasicula* group. Sampling material from cliff-dwelling individuals is crucial to capture the whole genetic variability of each taxon. This could enable the identification of evolutionarily significant units and genetic diversity hotspots, ultimately supporting future *in-situ* and *ex-situ* conservation projects.

Our study highlights the fact that UAS are tools that allow a significant refinement of demographic estimates, calculating more precise EOO and AOO. In particular, the role of the Z dimension, i.e. the cliff inclination, is of paramount importance when dealing with plant species tied to rupestrial habitats, and ignoring this fact may lead to a sensitive underestimation of the AOO.

Studies on population genetics, pollination biology and seed dispersal would be invaluable to assess the potential threat of fragmentation, especially given the reduced fecundity previously reported in this species (Commission of the European Communities 2009).

Furthermore, enhancing our knowledge about the physical, chemical, and biological properties of the soils where the pentamerous heathers grow is proving to be crucial. For instance, soil microbiota may host mycorrhizae useful for inoculation that may facilitate the establishment of the saplings during plant translocation initiatives.

*Ad hoc* research on reproductive biology, namely on plant-insect interactions (pollinators, predators), seed dispersal strategies and seedling or sapling establishment rates, is needed to address the problems of low reproductive fitness likely affecting all populations of the species.

As for *ex situ* conservation, the first known attempt to cultivate *E.sicula* outside its native range has been carried out at Kew (T. Freeth, pers. comm.). In 1952 the Royal Botanic Gardens hosted a plant grown in open ground, whose provenance and identity remain uncertain. In 1984, Kew hosted many individuals of E.siculasubsp.sicula grown from seeds collected by J.J. Archibald near Kemer (Türkiye). These plants, cultivated in pots, alpine houses, and more recently on the rock garden once their hardiness in UK climate was assured, have persisted through hostile weather conditions, including warm, wet, regularly frozen winters, and long summer droughts (T. Freeth, pers. comm.).

Along with the above-mentioned multifaceted research needed, some concrete interventions should be carried out as soon as possible. For instance, considering the clear preference of *Ericasiculas. l.* for shady and humid habitats, the increasing frequency of extreme heat, drought events, and wildfires in the coming decades will probably affect the survival of this group across its entire distribution range (MedECC Members 2019). Hence, to fight against the ongoing decline of many subpopulations, translocation initiatives should be encouraged (e.g., Abeli et al. 2021; Godefroid et al. 2025), especially in Italy, Libya, Lebanon, and Cyprus, where local disturbances are expected to persist or intensify in the next future.

In the specific case of E.siculasubsp.sicula, much more efforts should made to prevent and fight against the spread of invasive alien plants like the tussock alien grass *Cenchrussetaceus*, rapidly spreading along the disturbed coastal areas of NW Sicily and getting every year closer to Mt. Cofano (Pasta et al. 2010).

Contacts with the large number of scientists involved in this study were greatly facilitated by networks of Mediterranean specialists such as the IUCN/SSC/Mediterranean Plant Specialist Group and GENMEDA (http://www.genmeda.net/members/current_members/iucn). For this purpose, this study represents a paradigmatic case of international collaboration. Similar efforts should be encouraged and multiplied to strengthen the relationship between countries and improve knowledge, data sharing and produce effective and long-lasting conservation policies, going beyond the political and admi­nistrative borders of the countries hosting broad-ranged endangered plants (Pirie et al. 2022, 2024; Elliott et al. 2024).

## ﻿Additional information

### Author ORCIDs

Salvatore Pasta https://orcid.org/0000-0003-3265-9072

Hicham El Zein https://orcid.org/0000-0001-7541-9509

Ozan Şentürk https://orcid.org/0000-0002-2206-3745

Leopoldo de Simone https://orcid.org/0000-0002-3055-136X

### Data availability

All of the data that support the findings of this study are available in the main text or Supplementary Information.

## Supplementary materials

Supplementary material 1 Detailed information retrieved from the literature and from the labels of the 212 herbarium specimens examined Data type xlsx https://binary.pensoft.net/file/1282942This dataset is made available under the Open Database License (http://opendatacommons.org/licenses/odbl/1.0/). The Open Database License (ODbL) is a license agreement intended to allow users to freely share, modify, and use this Dataset while maintaining this same freedom for others, provided that the original source and author(s) are credited.Salvatore Pasta, Hicham El Zein, Ozan Şentürk, Salih Gücel, Leopoldo de Simone, Bertrand de Montmollin
